# Experiences from coordinating research after the 2011 terrorist attacks in Norway

**DOI:** 10.3402/ejpt.v5.23215

**Published:** 2014-07-02

**Authors:** Nils O. Refsdal

**Affiliations:** The Norwegian National Committees for Research Ethics, Oslo, Norway

**Keywords:** Disaster, research ethics, data sharing, terrorism, disaster research

## Abstract

This brief report presents some of the lessons learned from coordinating research in which people directly affected by terrorist attacks in Norway in 2011 are taking part. After the terrorist attacks, it was decided to establish a national coordinating function in order to protect those who were affected when they participate in research. By gathering key stakeholders, it is possible to avoid duplication of research through practical measures such as information sharing, facilitating cooperation, and working toward sharing of data. In addition, a coordinating function provides a platform for working to increase the impact of the research among practitioners and policy makers, and inform the general public. The conclusions are that coordination should be interdisciplinary, that it is important to plan for the sharing and reuse of data, and that both the research community and the research infrastructure should take steps to improve preparedness when disaster inevitably strikes again.

Not long after the terrorist attacks in Norway in July 2011, it became evident that there were several researchers who wanted to conduct research related to the terrorist attacks, particularly within health research. The South-Eastern Norway Regional Health Authority (Helse Sør-Øst) flagged the need to coordinate research not only to avoid duplication and an unnecessary strain on those who were affected but also to maximize the potential benefit from the research (Norwegian Directorate of Health, 2011). The Ministry of Health decided that the National Committees for Research Ethics in Norway would handle the coordinating function. A coordinator was appointed in August 2012 and the coordinating group was appointed shortly after. In this brief report, written by the coordinator, the work of the group will be presented, and some of the lessons we have learned pointed out.

## The need for coordinating research

Research after a disaster is ethically challenging. When involving disaster survivors in research, there may be an increased risk of emotional stress, although, at least in the field of psychotraumatology, the benefits for participants outweigh the negative effects when the researchers are properly trained, and the research design is methodologically and ethically sound (Newman & Kaloupek, 2009; Omerov, Steineck, Dyregrov, Runeson, & Nyberg, 2013). Disasters such as the terrorist attacks in Norway are public events, and survivors and the bereaved receive attention from the media, investigators, and researchers. This may affect consent decisions, either by making potential participants reject an enquiry by default (consent fatigue) or by their consenting to take part in studies without actually considering what they are consenting to (routinization of consent) (Ploug & Holm, 2013). Involving perpetrators may lead to other challenges, including legitimizing extremist ideology, misuse of such research by future terrorists, censorship by the security community, and the responsibility of balancing respondents’ confidentiality with the health and security of the public (Jones & Bhui, 2008; Kjos, 2013).

These are all challenges that must be dealt with for each individual research project. In this text, I concentrate on avoiding duplication of research and enquiries by researchers, which are challenges that must be handled by the infrastructure around these research projects. Duplication of research and contacts by researchers are both an unnecessary additional strain on respondents and a waste of resources. The attacks in Norway have generated a lot of research activity across academic fields. Researchers from the humanities and social sciences immediately started to analyze the perpetrator's manifesto, and social scientists, psychologists, and medical researchers started empirical research.^1^

Shortly after the attacks, the Norwegian Centre for Violence and Traumatic Stress Studies (NKVTS) started research projects involving survivors and their families, and the Centre for Crisis Psychology started research on the bereaved from the Utøya massacre. All of the survivors and most of the bereaved are involved in longitudinal studies that gather both quantitative data in the form of a wide range of psychological instruments and qualitative data in the form of free narratives of their experience of the bombing or the massacre, and the aftermath. Oslo University Hospital gathered data from the treatment of the injured for research, quality assurance, and documentation. They also undertook a survey of medical and rescue personnel, the police, and military staff who were involved as well as organized and spontaneous volunteers. There are also a handful of other projects that involved interviewing and conducting medical examinations of smaller groups of survivors and personnel. In sum, almost all of those who were affected have been approached at least once.

When researchers from different academic disciplines are interested in studying the same groups, the separate approval systems for different disciplines are a challenge. Medical research must have prior ethical approval from the Regional Committees for Medical and Health Research Ethics (REC).^2^ Other projects are required to notify the Norwegian Social Science Data Services (NSD).^3^ This means that no single institution assesses all projects and that no one sees the whole picture. When there is no formal cooperation or exchange of information between the two systems, there is a risk of duplicate research and requests for participation.

## The Norwegian coordination effort

The coordinating group was appointed in mid-2012. The coordinating effort is temporary and lasts until the end of 2014, with a possibility of extending the mandate if needed. The coordinating group is composed of key stakeholders representing research institutions, funding agencies, state and municipal authorities, and the support group. The objective is to safeguard the interests of those who were directly affected by the attacks when they participate in research. The tasks set out in the mandate are to monitor the load on the informant group, maintain an overview of ongoing and planned research activities, contribute to the exchange of information between researchers, and build networks and create meeting places.^4^ Funding and ethical approval follow normal procedures, and the coordination should not create additional bureaucracy for researchers.

The coordination is based on voluntary cooperation and exchange of information. The group does not approve or fund projects, or dictate the research agenda, and the researchers who are members of the coordinating group do not hold a monopoly on access to those who were affected. This is in contrast to the coordination undertaken at the University of Oklahoma after the 1995 bombing of the Alfred P. Murrah Federal Building. In that case, mental health research projects involving those affected were routed to the Institutional Review Board of the University of Oklahoma Health Sciences Center for ethical assessment. The research objectives were also identified centrally (Collogan, Tuma, Dolan-Sewell, Borja, & Fleischman, 2004). In order for the approach chosen in Norway to work, the members of the group must be able to influence decisions in their respective organizations. In addition, a high degree of trust and acknowledgement of the common goal is needed.

## Avoiding duplication

### Obtaining an overview

At the outset, no single institution had an overview of ongoing and planned research activities, making it difficult to assess the strain on respondents. Knowledge of which researchers ask what questions to what groups is essential to avoid duplication and facilitate cooperation. The most important funding agencies and research institutions are part of the coordinating group, which was helpful in getting an overview of planned and ongoing research. Routines for exchanging information with the Regional Committees for Medical and Health Research Ethics (REC) and the Norwegian Social Science Data Services (NSD) were also established. This has made it possible for REC and NSD to take the load on the informants into account in their decision-making.

### Facilitating cooperation

A coordinator with full knowledge of research activities lowers the threshold for cooperation across academic fields. Making researchers aware of each other and encouraging cooperation has been fruitful and has averted duplicate research and enquiries. Such cooperation can take different forms.

#### Splitting populations

Two projects planned to invite the same group of survivors from the Utøya massacre to a project where among others things they would undergo an fMRI scanning procedure. The researchers agreed to split the population and collect some data on behalf of each other and pool other data. Early clarifications on rights to data and a mutual understanding of who publishes what, and when, were prerequisites for this cooperation.

#### Cooperating on data collection logistics

Independent of the coordination work, researchers from Oslo University Hospital and the Norwegian Centre for Violence and Traumatic Stress Studies have coordinated recruitment and data collection from very early on. The purpose is to reduce the participants’ contact with researchers. Once again, it is crucial that formal agreements regulating the exchange and use of data are in place for such cooperation to work.

#### Inclusion of variables on behalf of others

By including questions from other researchers in an ongoing study, researchers may assist each other, so that multiple research groups may receive the information they need without conducting several interviews or surveys.

#### Sharing of data

Outright sharing of raw data between institutions has not, as far as we know, taken place. The consent forms and permissions given prevent this in most cases, since it was not planned for. Such sharing may be planned for, though, and should be considered in future disaster research.

#### Use of alternative data sources or populations

A good overview of ongoing research and data also makes it possible to suggest alternative sources of data. Examples include using the film from the trial rather than interviewing survivors about their reactions in court or using testimony given to the fact-finding commission rather than interviewing personnel. Some researchers have been advised to use other and more low profile populations that may work just as well for their purposes.

### Avoiding future enquiries and facilitating future research

The coordinating group is working to make data available for future research in a way that is methodologically sound, ethical, and consistent with the respondents’ wishes. In the short term, this means ensuring that data and metadata are well documented, and that data are not deleted. In the longer term, the group is exploring the possibility of collecting the data after researchers have finished with them, and storing them with a third party. Such a collection of data would be advantageous both for the research community and the participants themselves. For the research community, it would enable truly longitudinal studies, and also reduce the cost of data collection. Data could be reused not only within the field of psychotraumatology but also for research in other fields such as disaster management, disaster medicine, special pedagogy, and the sociology of law. For the participants, reuse means that they will receive fewer inquiries from researchers.

There are many obstacles to storing data in this manner. First, some researchers have planned to store data themselves for 20 years, and have consent forms and permits reflecting this. Others have a much shorter horizon and corresponding consent forms and permits. This means that consent forms and permits must be harmonized. Second, most of the data collection is publicly funded, which means that it should be made accessible to other researchers in an appropriate form after the original researcher are done with them. A minority of the data collection, however, is privately funded, and could be considered the intellectual property of the institution or the funders. Finally, it must be decided where the data will be stored and who gets access under what conditions. Some of these obstacles would have been easier to overcome if such storing was planned for from the outset.

## Maximizing the potential for learning

An added benefit of coordinating research is that it provides an opportunity for promoting good use of that research. In this section, I introduce some of the efforts undertaken by others and myself in this regard.

### Stakeholder involvement in research

Dialog between researchers and those who participate in research can be mutually beneficial. In its work, the National Support Group may receive information about the situation of the bereaved and what may have a positive impact on it. This can then be used for generating hypotheses that can be tested empirically by researchers. Likewise, the Support Group and the Workers’ Youth League may seek advice from researchers when planning visits to Utøya or in the potentially controversial processes of establishing memorials. Health services and central and municipal authorities have a responsibility for turning the knowledge produced by research into practice. Their involvement is important in order for research to have an impact on clinical practice, the organization of services and the information flow between administrative levels and between the authorities and practitioners.

### Dissemination

In order to communicate with their respondents and put the long-term follow-up of survivors and the bereaved on the public agenda, some of the projects have published “mini-reports” aimed at the respondents themselves and the general public (Dyb & Alve Glad, 2013; Dyb, Alve Glad, & Aadnanes, 2012; Dyregrov, Kristensen, & Johnsen, 2013). These are written in non-academic language and highlight key findings. The coordinating group has hosted breakfast seminars where researchers present their projects to the public and provide funding for conferences aimed at practitioners and experts.

### Guidance

The Support Group and the Workers’ Youth League are almost always asked to comment on research when it is presented in news media. For these groups, it is better to be briefed about a research project by someone directly involved in it or by a coordinator, than by a reporter who might have limited knowledge about the research. Funders, researchers, and students need guidance as well. Providing an overview of who asks what questions to whom, and where what data can be found, is valuable both to researchers and students who are in the early stages of a project. This keeps the research front moving forward.

### Providing an arena for ethical discussion

An added benefit of gathering researchers and stakeholders in a coordinating group is that it can provide an arena for debate and discussion about relevant ethical issues. We have hosted breakfast seminars on the use of the Internet as a source in the research after the attacks and on using extremists and terrorists as research participants.

The coordinating group has also invited about 20 PhD students working with the attacks to network meetings in order to provide them with an arena for discussing methods and research ethics. Some work alone and find it useful to discuss their projects with others working on the same subject matter, while others are part of larger projects where more experienced colleagues and supervisors have made methodological choices and ethical considerations before the PhD students became involved. In both cases, a network like this may be a useful arena for discussing ethical issues across academic fields.

### Preparing for the future

The group is trying to identify ways in which the research community can be better prepared when disaster strikes again. We have discussed how some research institutions may be singled out based on their research competence and national responsibility, for example, severe burns, epidemics, or psychological trauma. These institutions could prepare general and adaptable protocols based on relevant scenarios and work to reach a consensus on the use of measurements and instruments. The ethical approval system should be familiar with these protocols and have procedures to process applications based on them at short notice when necessary. The development of common protocols was also recommended after the coordination of research in the aftermath of the Oklahoma City bombing (Tassey, 1998). We will also raise the issue on how funding can be provided for early data collection to the funding agencies. Finally, some institutions have made agreements regulating the pooling, sharing, and reuse of data. Parts of these agreements could be reused, so that such cooperation can be in place from the start.

## Effects of the coordination effort and lessons learned

It is possible to meet some of the ethical challenges that arise in disaster research through practical measures. So far, duplication has been avoided through exchange of information and by lowering the threshold for cooperation. In addition, students, policy makers, researchers, and the general public have a better overview of the ongoing research than they would have had otherwise. Finally, the coordinating group has taken steps toward a possible data repository, which could become a unique collection of data for future research across academic fields.

In its final report, the American Psychological Association Task Force on the Mental Health Response to the Oklahoma City Bombing made five recommendations regarding research: (1) designation of a coordinating institution, (2) use of common research protocols, (3) study of long-term consequences, (4) comparison of treatment efficacy, and (5) establishing a mechanism for the funding of research (Tassey, 1998). The Norwegian experiences support these recommendations, with some additional points. First, when the need to coordinate research arises, it is important to designate the task early, preferably much earlier than in the Norwegian case. Second, it is important to have a clear mandate and to build trust between research groups, the infrastructure and the respondents themselves. Third, the Oklahoma coordination was limited to mental health research, but the Norwegian experience shows that a holistic and interdisciplinary point of departure is needed. Fourth, in order to make the study of long-term consequences and comparison of treatments possible, it is important to clarify the need for sharing and reusing data. This is also important to make sure that participants are not approached over and over. Finally, through the development of common disaster protocols and funding mechanisms, society can be better prepared, and make sure we learn as much as possible from future disasters.

## Footnotes

1 An overview of ongoing research and a bibliography is available here: http://www.etikkom.no/july22.

2 More about the procedure can be found here: https://helseforskning.etikkom.no/ikbViewer/page/forside?_ikbLanguageCode=us

3 More information about the Norwegian Social Science Data Services can be found here: http://www.nsd.uib.no/nsd/english/

4 My translation of the mandate given by The Ministry of Health. The full text of the mandate is available at http://www.etikkom.no/july22.
